# Predictors of Clinical Outcomes Among People With Human Immunodeficiency Virus and Tuberculosis Symptoms After Rapid Treatment Initiation in Haiti

**DOI:** 10.1093/ofid/ofaf031

**Published:** 2025-01-20

**Authors:** Aaron Richterman, Nancy Dorvil, Vanessa Rivera, Heejung Bang, Patrice Severe, Kerylyne Lavoile, Samuel Pierre, Alexandra Apollon, Emelyne Dumond, Guyrlaine Pierre Louis Forestal, Vanessa Rouzier, Patrice Joseph, Pierre-Yves Cremieux, Jean W Pape, Serena P Koenig

**Affiliations:** Department of Medicine (Infectious Diseases), University of Pennsylvania Perelman School of Medicine, Philadelphia, Pennsylvania, USA; Haitian Group for the Study of Kaposi’s Sarcoma and Opportunistic Infections (GHESKIO), Port-au-Prince, Haiti; Haitian Group for the Study of Kaposi’s Sarcoma and Opportunistic Infections (GHESKIO), Port-au-Prince, Haiti; Davis School of Medicine, University of California, Davis, California, USA; Haitian Group for the Study of Kaposi’s Sarcoma and Opportunistic Infections (GHESKIO), Port-au-Prince, Haiti; Haitian Group for the Study of Kaposi’s Sarcoma and Opportunistic Infections (GHESKIO), Port-au-Prince, Haiti; Haitian Group for the Study of Kaposi’s Sarcoma and Opportunistic Infections (GHESKIO), Port-au-Prince, Haiti; Haitian Group for the Study of Kaposi’s Sarcoma and Opportunistic Infections (GHESKIO), Port-au-Prince, Haiti; Haitian Group for the Study of Kaposi’s Sarcoma and Opportunistic Infections (GHESKIO), Port-au-Prince, Haiti; Haitian Group for the Study of Kaposi’s Sarcoma and Opportunistic Infections (GHESKIO), Port-au-Prince, Haiti; Haitian Group for the Study of Kaposi’s Sarcoma and Opportunistic Infections (GHESKIO), Port-au-Prince, Haiti; Department of Medicine, Weill Cornell Medical College, New York, New York, USA; Haitian Group for the Study of Kaposi’s Sarcoma and Opportunistic Infections (GHESKIO), Port-au-Prince, Haiti; Analysis Group, Boston, Massachusetts, USA; Haitian Group for the Study of Kaposi’s Sarcoma and Opportunistic Infections (GHESKIO), Port-au-Prince, Haiti; Department of Medicine, Weill Cornell Medical College, New York, New York, USA; Brigham and Women's Hospital, Harvard Medical School, Boston, Massachusetts, USA

**Keywords:** Haiti, HIV, outcome predictors, rapid antiretroviral therapy initiation, low- and middle-income countries

## Abstract

**Background:**

Few studies have evaluated baseline predictors of clinical outcomes among people with human immunodeficiency virus (HIV) starting antiretroviral therapy (ART) in the modern era of rapid ART initiation.

**Methods:**

We conducted a secondary analysis of a previously reported open-label randomized controlled trial of 2 rapid treatment initiation strategies for people with treatment-naive HIV and tuberculosis symptoms at a large urban clinic in Haiti. We used logistic regression models to assess associations between baseline characteristics and (1) retention in care at 48 weeks, (2) HIV viral load suppression at 48 weeks (among participants who underwent viral load testing), and (3) all-cause mortality. For the viral load suppression outcome, we used inverse probability weighting to account for potential selection bias resulting from exclusion of participants who did not undergo viral load testing.

**Results:**

A total of 500 participants were enrolled in the study from November 2017 to January 2020. Tuberculosis was diagnosed in 88 participants (18%), and ART was started in 494 (99%). After multivariable adjustment, less than secondary school education (adjusted odds ratio [AOR] 0.21 [95% confidence interval (CI), .10–.46]) was significantly associated with a reduced odds of retention in care. Dolutegravir initiation (AOR, 2.57 [95% CI, 1.22–5.43]), age (1.42 per 10-year increase [1.01–1.99]), and tuberculosis diagnosis (3.92 [1.36–11.28]) were significantly associated with increased odds of retention. Age (AOR, 1.36 [95% CI, 1.05–1.75]) and dolutegravir initiation (1.75 [1.07–2.85]) were positively associated with viral suppression, and tuberculosis diagnosis (0.50 [.28–.89) was negatively associated with viral suppression, with similar findings after incorporation of inverse probability weights. Higher CD4 cell count at enrollment was significantly associated with a lower odds of mortality (unadjusted odds ratio, 0.69 [95% CI, .55–.87]), and anemia was associated with a significantly greater odds of mortality (4.86 [1.71–13.81]).

**Conclusions:**

We identified sociodemographic, treatment-related, clinical, and laboratory-based predictors of clinical outcomes. These characteristics may serve as markers of subpopulations that could benefit from additional interventions to support treatment success after rapid treatment initiation.

## Teaser

This secondary analysis of a randomized controlled trial of rapid treatment initiation strategies for people with treatment-naïve HIV and tuberculosis in Haiti identified sociodemographic, treatment-related, clinical, and laboratory-based predictors of clinical outcomes after rapid antiretroviral therapy initiation.

Rapid initiation of antiretroviral therapy (ART) after human immunodeficiency virus (HIV) diagnosis (ie, within 7 days of diagnosis, including same-day start) has been recommended by the World Health Organization (WHO) since 2017 [1]. The 2021 WHO guidelines recommend rapid initiation for all people with HIV except for those with concern for meningitis (ie, secondary to cryptococcus or tuberculosis) [2]. This recommendation was based on multiple randomized controlled trials conducted in diverse settings and published over the last decade [3–9]. These trials demonstrated that rapid ART initiation results in improvements in retention in care, viral load suppression, and mortality. There have been 2 hypothesized mechanisms behind this benefit—first, that logistical burdens are minimized; and second, that the more immediate provision of medicine facilitates a sense of hope, optimism, and connection with the health system. While several observational studies using real-world data have suggested that rapid ART initiation may be associated with increased risk of loss to follow-up, these findings can largely be attributed to biases inherent to the study design [10].

People with newly diagnosed HIV commonly present with signs or symptoms suggesting tuberculosis [11]. While the 2021 WHO guidelines acknowledged that there is little information available about the risks or benefits of rapid ART initiation among people with HIV and tuberculosis symptoms, they recommended rapid ART initiation while investigating tuberculosis, with close follow-up within 7 days to initiate tuberculosis treatment if necessary. Since the publication of these guidelines, our group has demonstrated high rates of ART initiation and retention in care among people with HIV and tuberculosis symptoms in Haiti who were randomized to either of 2 rapid treatment initiation strategies [12].

While the recently shifting approach in ART initiation timing reflects the clearly apparent benefits associated with a rapid start, this strategy necessitates a shorter window of time to allow for the assessment of clinical or social risk factors for poor outcomes after starting ART. While many studies have evaluated predictors of clinical outcomes among people with HIV starting ART, we are aware of none evaluating predictors of outcomes in the context of rapid ART initiation. As a result, a key research priority in the rapid ART initiation era is to identify readily available baseline characteristics associated with subsequent treatment outcomes such as retention in care, viral suppression, and mortality, including among people with HIV experiencing tuberculosis symptoms. A better understanding of these predictors may allow for the development of targeted interventions for subpopulations of people starting ART who are vulnerable to poor outcomes after rapid treatment initiation. To address this gap in the literature, we evaluated baseline predictors of clinical outcomes in a randomized trial of 2 rapid treatment initiation strategies for people with HIV and tuberculosis symptoms receiving care in urban Haiti [12].

## METHODS

### Study Design and Oversight

We conducted a secondary analysis of a previously reported open-label randomized controlled trial of treatment initiation strategies for people with treatment-naive HIV and tuberculosis symptoms in Haiti (NCT03154320) [12]. Our objective in this secondary analysis was to evaluate baseline predictors of (1) retention in care, (2) HIV viral load suppression at 48 weeks, and (3) all-cause mortality. Participants in the trial were randomized 1:1 to either same-day treatment (same-day tuberculosis testing with same-day tuberculosis treatment if tuberculosis was diagnosed or same-day ART if it was not) versus standard care (starting tuberculosis treatment within 7 days and delaying ART to day 7 if tuberculosis was not diagnosed). Tuberculosis diagnostics included chest radiography, Xpert Ultra assay (Cepheid), and liquid mycobacterial culture (mycobacteria growth indicator tube [MGIT]; BACTEC; Beckton Dickinson [BD]), with detailed diagnostic and treatment algorithms for the same-day and standard care groups, described elsewhere [12]. For all participants with a diagnosis of tuberculosis, ART was initiated 2 weeks after tuberculosis treatment. The trial found no differences by study arm in the primary outcome of retention in care with 48-week HIV-1 RNA levels <200 copies/mL. Additional details about the treatment strategies and outcomes have been reported elsewhere [12]. The trial was approved by the institutional review boards at the Haitian Group for the Study of Kaposi's Sarcoma and Opportunistic Infections (GHESKIO), Mass General Brigham, Florida International University, and Weill Cornell Medical College.

### Setting

The study was conducted at GHESKIO in Port-au-Prince, the urban, densely populated capital of Haiti. During the study there was pervasive and ongoing geopolitical instability, gang violence, and civil unrest in Haiti, with consequent economic instability and hardship. GHESKIO is a Haitian nongovernmental organization and the largest provider of HIV and tuberculosis care in the Caribbean. GHESKIO provides care for approximately 15 000 people with HIV and >2000 with tuberculosis annually. The adult HIV prevalence in Haiti is 1.7%, and the annual tuberculosis incidence is 154 per 100 000 persons [13, 14]. First-line treatment for HIV included coformulated efavirenz, tenofovir disoproxil fumarate, and lamivudine until December 2018, after which dolutegravir became available and was preferred over efavirenz. During 2019, all people with HIV receiving efavirenz-based regimens at GHESKIO were switched to a dolutegravir-based regimen, regardless of viral load. Tuberculosis was treated according to standard guidelines, and all participants without a diagnosis of tuberculosis disease were treated with prophylactic isoniazid [15]. Based on recent clinical trial results, participants with CD4 cell counts <100/μL received azithromycin for 5 days, and those with counts <100/μL *and* tuberculosis received prednisone prophylaxis [16, 17]. All participants received trimethoprim-sulfamethoxazole prophylaxis on the day of HIV diagnosis, regardless of CD4 cell count. Retention activities at GHESKIO included reminder phone calls (or home visits for those without phones) before visits and after missed visits, a transportation subsidy of approximately $1 (US dollars throughout) at each visit, and a phone card for approximately $1 at each visit.

### Study Population

We included all participants enrolled in the original trial in this secondary analysis [12]. Patients were eligible for inclusion in the trial if they had documented HIV-1 infection, were ≥18 years of age and ART naive, and reported cough, fever, night sweats of any duration, and/or weight loss. Exclusion criteria included tuberculosis treatment in the past year, lack of preparedness on an ART readiness questionnaire, pregnancy or breastfeeding, active drug or alcohol use, a mental condition that would interfere with completing study requirements, plans to transfer during the study period, symptoms consistent with WHO stage 4 neurologic disease, or WHO “danger signs” of temperature >39°C, pulse rate >120/min, respirations >30/min, or inability to walk unaided. All participants provided written informed consent.

### Study Data

Study procedures have been reported in detail elsewhere [12]. For the purposes of this analysis, we included baseline demographic, clinical and laboratory data, as well as outcome data 48 weeks after enrollment, all of which were extracted from the GHESKIO electronic health record. Baseline variables included in this analysis were: age, sex, income <$1 per day (self-report), educational attainment, marital status, body mass index (BMI), CD4 cell count, tuberculosis diagnosis (microbiologically or clinically diagnosed), and study arm (same-day treatment or standard care). We also included information about the ART regimen that was initiated. Outcome variables included clinic attendance at 48 weeks, HIV-1 RNA at 48 weeks, and mortality during 48 weeks of follow-up.

### Outcomes

We considered 3 outcomes in this analysis. Retention in care was defined as attending a clinical visit 48 weeks after enrollment (with a prespecified window of ±2 weeks) and was evaluated among all participants who did not transfer care. Viral load suppression was defined as having an HIV-1 RNA level <200 copies/mL 48 weeks after enrollment (with a prespecified window of ±12 weeks) and was evaluated among all participants who had undergone viral load testing within that window. All-cause mortality over 48 weeks of follow-up after enrollment was determined using family report (death certificates are not available in Haiti) and was evaluated among all participants who did not transfer care. Participants who died were defined as not retained in care and not suppressed at week 48.

### Statistical Analysis

Baseline characteristics were summarized using medians and interquartile ranges (IQRs) for continuous variables and frequencies and percentages for categorical/binary variables. We assessed predictors of retention in care, viral suppression, and mortality using logistic regression models with a complete case analysis. We evaluated associations between our outcome variables and the following baseline predictors: age (continuous variable, per 10-year increase), female sex, income <$1 per day, less than secondary school education, married status, undernutrition (BMI <18.5 [calculated as weight in kilograms divided by height in meters squared]), CD4 cell count (continuous variable, per 50/μL increase), anemia (National Institutes of Health Division of AIDS grade ≥3; with missing values considered not anemic) [18], tuberculosis diagnosis, and randomization to the standard strategy study arm. Because first-line ART changed from an efavirenz-based to a dolutegravir-based regimen partway through the study, we also included a binary variable indicating whether a participant had been started on dolutegravir. We prespecified this set of 11 predictors and cutoffs for binary predictors from clinical perspectives; we did not conduct model-based variable selection. We did not adjust time-varying covariates that may be less relevant for prediction or variables that can be affected by study treatment/exposure (eg, symptoms during follow-up).

We first evaluated univariable associations between predictors and the 3 outcomes. We then generated multivariable models for the retention in care and viral load suppression outcomes that included all predictors. We did not build a multivariable model for the mortality outcome because there were few deaths (n = 15).

Next, for the viral load suppression outcome, we also fitted an inverse probability of (censoring) weighting regression model to account for potential selection bias resulting from exclusion of 69 participants who did not undergo viral load testing at week 48 [19, 20]. To do this, we weighted observations by the inverse probability that a given participant had a viral load test performed. These probabilities of having nonmissing (or complete) data were fitted via multivariable logistic regression that included the 11 baseline covariates in this analysis [20]. As a secondary analysis, we repeated the viral load suppression analysis among all participants, with participants who did not complete week 48 viral load testing considered nonsuppressed. We performed statistical analyses using SAS software, version 9.4 (SAS Institute).

## RESULTS

Of 576 people screened for trial participation between November 2017 and January 2020, 500 were enrolled and are included in the current analysis (Table 1) [12]. Their median age (IQR) was 37 years (30–45) years, and 234 (47%) were female. Cough, fever, night sweats, and weight loss were reported by 200 (47%), 194 (39%), 72 (14%), and 491 (98%) of the participants, respectively. The median BMI (IQR) was 20.6 (18.7–22.9), and 116 participants (23%) met criteria for undernutrition. The median hemoglobin level (IQR) was 11.8 (10.1–11.8) g/dL, and 81 participants (16%) with available hemoglobin results (n = 480) had grade ≥3 anemia [18]. The median CD4 cell count (IQR) was 274/μL (I28–426/μL), with 101 (20%) having a count <100/μL. Eighty-eight participants (18%) had tuberculosis diagnosed at baseline; of these tuberculosis cases, 68 (77%) were diagnosed microbiologically (ie, positive Xpert). All participants with tuberculosis were started on first-line treatment for drug-susceptible tuberculosis.

**Table 1.** Baseline Characteristics of Study Participants

Characteristic	Participants, No (%)^a^ (n = 500)
Age at enrollment, median (IQR), y	37 (30–45)
Female sex	234 (47)
Income <$1/d (USD)	372 (74)
Less than secondary school education	192 (38)
Married status	261 (52)
BMI, median (IQR)^b^	20.6 (18.7–22.9)
Undernutrition (BMI <18.5)^b^	116 (23)
CD4 cell count, median (IQR), cells/μL (n = 495)	274 (128–426)
Hemoglobin, median (IQR), g/dL (n = 480)	11.8 (10.1–13.4)
Anemia (grade ≥3)^c^ (n = 480)	81 (16)
Tuberculosis diagnosed at enrollment	88 (18)
Dolutegravir-based ART initiated (vs efavirenz; among ART initiators) (n = 494)	184 (37)
Randomized to standard (vs same-day) treatment group	250 (50)

Abbreviations: ART, antiretroviral therapy; BMI, body mass index; IQR, interquartile range; USD, US dollars.

^a^Data represent no. (%) of participants unless otherwise specified (n = 500 unless otherwise specified).

^b^BMI calculated as weight in kilograms divided by height in meters squared.

^c^The National Institutes of Health Division of AIDS threshold for anemia of grade ≥3 severity is hemoglobin value ≤9 g/dL for male and ≤8.5 g/dL for female individuals.

ART was started in participants 494 (99%)—407 (83%) without tuberculosis diagnosed at baseline, 69 (14%) who started ART after starting tuberculosis treatment, and 18 (4%) who started ART before starting tuberculosis treatment (eg, in the context of positive culture after initial negative Xpert result). Among participants who started ART, 310 (62%) started an efavirenz-based regimen and 184 (37%) a dolutegravir-based regimen. Among the 412 participants without tuberculosis diagnosed at baseline, ART was initiated in 407 (98.8%) at a median time (IQR) of 6 (0–7) days—on the day of HIV diagnosis in 202 (49%), within 7 days in 110 (27%), 8–14 days after HIV diagnosis in 83 (20%), and >14 days after diagnosis in 12 (3%). Among participants started on efavirenz, 158 (51%) were transitioned to dolutegravir during the study period, a median (IQR) of 245 (201–289) days after ART initation. Nine participants (2%) who were started on an efavirenz-based regimen were switched to a second-line protease inhibitor-based regimen during the study period.

Of 447 participants (89%) retained in care at week 48, 431 (96%) underwent viral load testing. Among those who underwent viral load testing, 320 (72%) had HIV-1 RNA levels <200 copies/mL. Fifteen participants (3%) died during the study period, with causes of death reported elsewhere [12].

In univariate analyses, less than secondary school education (odds ratio [OR], 0.34 [95% confidence interval (CI), .17–.66]) was associated with a reduced odds of retention in care at 48 weeks, and initiation of dolutegravir-based ART (2.74 [1.34–5.60]) was significantly associated with an increased odds of retention in care (Table 2). After multivariable adjustment, less than secondary education (adjusted OR [AOR], 0.21 [95% CI, .10–.46]) remained significantly associated with a reduced odds of retention in care. Dolutegravir initiation (AOR, 2.57 [95% CI, 1.22–5.43]), age (1.42 per 10-year increase [1.01–1.99]), and tuberculosis diagnosis at enrollment (3.92 [1.36–11.28]) were all significantly associated with increased odds of retention.

**Table 2.** Baseline Predictors of Retention in Care at 48 Weeks (n = 500)

Predictor	Univariable Regression		Multivariable Regression^a^	
	OR (95% CI)	*P* Value	OR (95% CI)	*P* Value
Age (per 10-y increase)	1.19 (.88–1.61)	.25	1.42 (1.01–1.99)	.04
Female sex	0.98 (.56–1.74)	.95	1.28 (.67–2.43)	.46
Income <$1/d (USD)	0.84 (.42–1.64)	.60	0.80 (.37–1.70)	.55
Less than secondary school education	0.34 (.17–.66)	.001	0.21 (.10–.46)	<.001
Married status	0.82 (.46–1.46)	.50	0.72 (.38–1.35)	.30
Undernutrition	1.04 (.53–2.04)	.92	0.97 (.46–2.05)	.93
CD4 cell count at enrollment (per 50/μL increase)	1.01 (.95–1.08)	.67	1.04 (.97–1.10)	.30
Anemia (grade ≥3)^b^	0.81 (.39–1.69)	.58	0.51 (.23–1.16)	.11
Tuberculosis diagnosed at enrollment	2.19 (.85–5.67)	.11	3.92 (1.36–11.28)	.01
Randomized to standard (vs same-day) treatment group	1.60 (.90–2.87)	.11	1.73 (.93–3.25)	.09
Dolutegravir-based ART	2.74 (1.34–5.60)	.006	2.57 (1.22–5.43)	.01

Abbreviations: ART, antiretroviral therapy; CI, confidence interval; OR, odds ratio; USD, US dollars.

^a^Data were missing in 5 persons (area under the receiver operating characteristic curve, 0.75).

^b^The National Institutes of Health Division of AIDS threshold for anemia of grade ≥3 severity is hemoglobin value ≤9 g/dL for male and ≤8.5 g/dL for female individuals.

Among participants who underwent viral load testing at 48 weeks, age (OR, 1.35 per 10-year increase [95% CI, 1.08–1.71]) and dolutegravir initiation (1.81 [1.13–2.90) were significantly associated with an increased odds of viral suppression; tuberculosis diagnosis (0.42 [.25–.69]) was associated with a reduced odds of viral suppression (Table 3). After multivariable adjustment, age (AOR, 1.36 [95% CI, 1.05–1.75]) and dolutegravir initiation (1.75 [1.07–2.85) remained positively associated with viral suppression, and tuberculosis diagnosis (0.50 [.28–.89) remained negatively associated with it. Findings were similar after incorporating inverse probability weights to account for participants who did not undergo viral load testing (Table 3). Weights ranged from 1.02 to 1.48, which means that 1 complete case/person represented approximately 1–1.5 person/observation from the overall population. In a secondary analysis of viral load suppression among all participants, with participants who did not complete week 48 viral load testing considered nonsuppressed, results were overall similar to our primary analysis except that tuberculosis diagnosis was no longer associated with viral suppression and less than secondary school education was now associated with a lower odds of viral load suppression (Supplementary Table 1). Higher CD4 cell count at enrollment was significantly associated with a lower odds of mortality in unadjusted models (OR, 0.69 [95% CI, .55–.87) (Table 4), and grade ≥3 anemia with a significantly greater odds of mortality (4.86 [1.71–13.81]).

**Table 3.** Baseline Predictors of Viral Suppression Among Participants With Human Immunodeficiency Virus 1 RNA Testing at 48 Weeks

Predictor	Univariable Analysis (n = 431)		Multivariable Analysis (n = 429)^a^		Multivariable Analysis With Inverse Probability Weights (n = 429)^b^	
	OR (95% CI)	*P* Value	OR (95% CI)	*P* Value	OR (95% CI)	*P* Value
Age (per 10-y increase)	1.35 (1.08–1.71)	.01	1.36 (1.05–1.75)	.02	1.35 (1.07–1.70)	.01
Female sex	1.05 (.68–1.62)	.82	1.05 (.66–1.67)	.84	1.05 (.68–1.62)	.83
Income <$1/d (USD)	0.90 (.55–1.49)	.69	1.11 (.64–1.90)	.72	1.10 (.66–1.82)	.72
Less than secondary school education	0.89 (.57–1.37)	.58	0.78 (.48–1.27)	.32	0.79 (.50–1.23)	.29
Married status	1.44 (.93–2.22)	.10	1.42 (.90–2.26)	.14	1.49 (.97–2.29)	.07
Undernutrition	0.70 (.43–1.14)	.15	0.89 (.25–1.54)	.68	0.87 (.52–1.44)	.58
CD4 cell count at enrollment (per 50/μL increase)	1.04 (.99–1.09)	.16	1.03 (.98–1.08)	.31	1.03 (.98–1.08)	.28
Anemia (grade ≥3)^c^	0.82 (.46–1.46)	.50	1.07 (.57–2.01)	.83	1.05 (.58–1.90)	.87
Tuberculosis diagnosed at enrollment	0.42 (.25–.69)	<.001	0.50 (.28–.89)	.02	0.50 (.29–.86)	.01
Randomized to standard (vs same-day) treatment group	1.25 (.81–1.93)	.31	1.15 (.73–1.81)	.54	1.11 (.73–1.69)	.63
Dolutegravir-based ART	1.81 (1.13–2.90)	.01	1.75 (1.07–2.85)	.03	1.72 (1.09–2.72)	.02

Abbreviations: ART, antiretroviral therapy; CI, confidence interval; OR, odds ratio; USD, US dollars.

^a^Data were missing in 2 persons (area under the receiver operating characteristic curve [AUC], 0.66).

^b^CIs were estimated assuming that weight is known, since very similar CIs were obtained when weight was known and when it was estimated (AUC, 0.66).

^c^The National Institutes of Health Division of AIDS threshold for anemia of grade ≥3 severity is hemoglobin value ≤9 g/dL for male and ≤8.5 g/dL for female individuals.

**Table 4.** Baseline Predictors of Mortality

Predictor	Univariable Analysis^a^	
	OR (95% CI)	*P* Value
Age (per 10-y increase)	1.25 (.76–2.06)	.38
Female sex	0.99 (.36–2.79)	.99
Income <$1/d (USD)	0.50 (.18–1.45)	.20
Less than secondary school education	1.59 (.54–4.73)	.40
Married status	0.60 (.21–1.72)	.34
Undernutrition	1.69 (.56–5.03)	.35
CD4 cell count at enrollment (per 50/μL increase)	0.69 (.55–.87)	.002
Grade ≥3 anemia^b^	4.86 (1.71–13.81)	.003
Tuberculosis diagnosed at enrollment	0.33 (.04–2.52)	.28
Randomized to standard (vs same-day) treatment group	0.66 (.23–1.88)	.43
Dolutegravir-based ART	0.62 (.19–1.96)	.41

Abbreviations: ART, antiretroviral therapy; CI, confidence interval; OR, odd ratio: USD, US dollars.

^a^We did not conduct multivariable regression because there were only 15 deaths.

^b^The National Institutes of Health Division of AIDS threshold for anemia of grade ≥3 severity is hemoglobin value ≤9 g/dL for male and ≤8.5 g/dL for female individuals.

## DISCUSSION

In this study of 500 people with newly diagnosed HIV and tuberculosis symptoms (with a tuberculosis diagnosis in about 1 in 5) who participated in a randomized trial of 2 rapid treatment initiation protocols, we identified sociodemographic (age, educational attainment), treatment-related (dolutegravir-based regimens), clinical (tuberculosis diagnosis), and laboratory-based (CD4 cell count and anemia) predictors of clinical outcomes over a follow-up period of 48 weeks. Despite substantial geopolitical instability in Haiti during the study period, nearly all participants started ART (more than three-quarters of participants without tuberculosis started within 7 days), and that rate of retention in care was high. This is one of the first studies to report baseline predictors of clinical outcomes in the context of rapid ART initiation, especially among people experiencing tuberculosis symptoms. Despite a rapid ART initiation strategy necessitating a shorter window of time to allow for the assessment of clinical or social risk factors for poor outcomes after initiating ART, we found similar predictors of clinical outcomes as in the era before rapid ART initiation. Importantly, the predictors we identified can be easily and quickly measured during the condensed evaluation before rapid ART initiation and may serve as markers of subpopulations that could benefit from additional interventions to support treatment success.

The 2 sociodemographic factors associated with poorer outcomes were younger age (associated with lower retention in care and lower viral suppression rates) and less than a secondary school education (associated with lower retention in care). Both of these factors have previously been identified as predictors of clinical outcomes in pre–rapid ART initiation settings [21], and our findings demonstrate that they continue to be important markers in the current era.

Educational level was the baseline characteristic most strongly associated with retention in care. It was also identified as a key driver of persistent disparities in life expectancy among patients starting ART at GHESKIO during earlier time periods [22]. Our results may reflect several potential mechanisms. First, lower educational level may have been associated with lower health literacy, with greater consequent difficulty communicating the benefits of ART and clinical follow-up. If this is the case, patients with lower education levels may benefit from tools designed to facilitate effective and rapid communication of key HIV-related concepts [23, 24]. While this may be exacerbated by the shorter duration of pretreatment counseling inherent to rapid ART initiation, as we noted above this association was also described before the adoption of rapid ART initiation [21]. Second, this association may result from an unmeasured social or economic factor that is correlated with education. For example, food insecurity tends to be strongly associated with educational attainment, is highly prevalent in Haiti, and has been associated with a wide variety of adverse HIV outcomes [25–28]. More generally, socioeconomic factors like poverty are associated with education and can strongly influence HIV outcomes [29]. While future research should aim to disentangle these potential mechanisms, educational level can be used in HIV programs as a prognostic marker for clinical outcomes after treatment initiation.

Initiation of a dolutegravir-based regimen (vs an efavirenz-based regimen) was associated with an increased odds of both retention in care and viral suppression. This finding further supports the preference for dolutegravir as first-line therapy [2], is consistent with dolutegravir's greater tolerability and effectiveness relative to efavirenz [30] and illustrates the instrumental role that dolutegravir can play in improving HIV-related population health. This is especially the case in settings with high rates of efavirenz resistance among ART-naive people with HIV, like Haiti [31]. Of note, about half the participants started on a efavirenz-based ART were transitioned to dolutegravir at some point during the study period. This likely biased the association between dolutegravir initiation and clinical outcomes toward the null, meaning that the benefits of dolutegravir are likely to be even greater than those estimated in this study. Of note, dolutegravir is now the standard of care for first-line ART in Haiti.

While all participants in this study were experiencing tuberculosis symptoms at enrollment, tuberculosis was ultimately diagnosed in only 18%, and a tuberculosis diagnosis was significantly associated with greater odds of retention in care and lower odds of viral suppression. Greater retention likely results from closer clinical contact during tuberculosis treatment (eg, directly observed therapy) and the fact that people with tuberculosis commonly experience rapid improvement in symptoms with therapy. Other studies have also reported an association between virologic failure and tuberculosis coinfection [32–35]. Lower odds of viral suppression after tuberculosis diagnosis may relate to delays in ART initiation (although these tended to be short) or difficulty with drug tolerability, given that additional medications are administered. Tuberculosis may also increase HIV viral load and the size of the HIV reservoir.

There were few deaths among participants during follow-up, and 2 baseline laboratory characteristics were significantly associated with death—CD4 cell count and anemia. Immunologic dysfunction from progressive HIV has long been recognized as a key risk factor for death among people with HIV. Anemia, which may result from both nutritional and nonnutritional (eg, disseminated infection) mechanisms, has also been identified as an important risk factor for mortality [36, 37].

This study had several limitations. It was conducted among participants reporting tuberculosis symptoms at a single large urban clinic with a high quality of care, which may limit generalizability to other settings and contexts. Our viral suppression outcome was assessed among participants who completed viral load testing at week 48, which raises the possibility of selection bias. However, >85% of participants underwent viral load testing, and estimates from our missing data-adjusted analyses were nearly identical to those from an unweighted multivariable model. Finally, the ORs reported here are associational, not casual, with nonrandomized exposures, which is typical in studies of identification of predictors or risk or protective factors.

In conclusion, in this study of 500 people with newly diagnosed HIV and tuberculosis symptoms (with tuberculosis diagnosed in 18%) who participated in a trial of 2 rapid treatment initiation protocols, we identified sociodemographic (age, educational attainment), treatment-related (dolutegravir-based regimens), clinical (tuberculosis diagnosis), and laboratory-based (CD4 cell count and anemia) predictors of clinical outcomes over a follow-up period of 48 weeks. This is one of the first studies to report baseline predictors of clinical outcomes in the context of rapid ART initiation, especially among people reporting tuberculosis symptoms. The predictors we identified can be easily and quickly assessed during the condensed evaluation before rapid ART initiation and may serve as markers of subpopulations that could benefit from additional interventions to support treatment success.

## Supplementary Material

ofaf031_Supplementary_Data

## Supplementary Data

Supplementary materials are available at *Open Forum Infectious Diseases* online. Consisting of data provided by the authors to benefit the reader, the posted materials are not copyedited and are the sole responsibility of the authors, so questions or comments should be addressed to the corresponding author.
