# Supplementary material for: Predictors of Clinical Outcomes Among People With Human Immunodeficiency Virus and Tuberculosis Symptoms After Rapid Treatment Initiation in Haiti
Source: Open Forum Infect Dis. 2025 Jan 20;12(2):ofaf031. doi: 10.1093/ofid/ofaf031 (PMC11793062; doi:10.1093/ofid/ofaf031)
Supplement: ofaf031_Supplementary_Data [file ofaf031_supplementary_data.docx]

**Supplementary Material For:**

**Predictors of Clinical Outcomes among People with HIV and Tuberculosis Symptoms after Rapid Treatment Initiation in Haiti**

^1^Aaron RICHTERMAN*, ^2^Nancy DORVIL*, ^2^Vanessa RIVERA, ^3^Heejung BANG, ^2^Patrice SEVERE, ^2^Kerylyne LAVOILE, ^2^Samuel PIERRE, ^2^Alexandra APOLLON, ^2^Emelyne DUMOND, ^2^Guyrlaine PIERRE LOUIS FORESTAL, ^2,5^Vanessa ROUZIER, ^2^Patrice JOSEPH, ^4^Pierre-Yves CREMIEUX, ^2,5^Jean W PAPE, ^6^Serena P KOENIG

*co-first authors

Corresponding author: Aaron Richterman; Hospital of the University of Pennsylvania, 3400 Spruce Street, Philadelphia, PA, USA (19104); e-mail: [aaron.richterman@pennmedicine.upenn.edu](mailto:aaron.richterman@pennmedicine.upenn.edu)

Alternative corresponding author: Serena Koenig; Brigham and Women’s Hospital, 75 Francis Street, Boston, MA, United States of America; [skoenig@bwh.harvard.edu](mailto:skoenig@bwh.harvard.edu)

^1^ University of Pennsylvania Perelman School of Medicine, Philadelphia, Pennsylvania, United States of America

^2^ Haitian Group for the Study of Kaposi’s Sarcoma and Opportunistic Infections (GHESKIO), Port-au-Prince, Haiti

^3^ University of California, Davis School of Medicine, Davis, California, United States of America

^4^Analysis Group, Boston, Massachusetts, United States of America

^5^ Weill Cornell Medical College, New York, New York, United States of America

^6^ Brigham and Women’s Hospital, Harvard Medical School, Boston, Massachusetts, United States of America

**Supplementary Table 1. Baseline Predictors of Viral Suppression among All Participants (i.e., missing week 48 viral load considered to be not virally suppressed).**

| Predictors | Univariable Analysis  (N=500) | | Multivariable Analysis  (N=495)** | |
| --- | --- | --- | --- | --- |
|  | OR (95% CI) | p-value | OR (95% CI) | p-value |
| Age (per 10 year increase) | 1.28 (1.06, 1.55) | 0.01 | 1.33 (1.08, 1.64) | 0.007 |
| Female sex | 1.04 (0.72, 1.51) | 0.19 | 1.10 (0.75, 1.63) | 0.63 |
| Income <$1 USD per day | 0.91 (0.60, 1.39) | 0.66 | 1.06 (0.67, 1.67) | 0.81 |
| Less than secondary education | 0.70 (0.48, 1.01) | 0.06 | 0.59 (0.39, 0.89) | 0.01 |
| Married | 1.23 (0.85, 1.77) | 0.27 | 1.18 (0.80, 1.74) | 0.40 |
| Undernutrition | 0.74 (0.49, 1.14) | 0.17 | 0.83 (0.52, 1.32) | 0.43 |
| CD4 count at enrollment (per 50 cells/mm^3^ increase) | 1.02 (0.98, 1.06) | 0.34 | 1.02 (0.98, 1.06) | 0.46 |
| Anemia (Grade ≥3)* | 0.84 (0.51, 1.36) | 0.47 | 0.93 (0.55, 1.57) | 0.93 |
| Diagnosed with tuberculosis at enrollment | 0.62 (0.39, 0.99) | 0.04 | 0.79 (0.47, 1.33) | 0.38 |
| Randomized to standard group (vs. same-day group) | 1.32 (0.92, 1.91) | 0.14 | 1.29 (0.88, 1.89) | 0.19 |
| Dolutegravir-based ART | 1.73 (1.17, 2.56) | 0.006 | 1.62 (1.08, 2.43) | 0.02 |

*NIH DAIDS threshold for anemia of Grade ≥3 severity is hemoglobin value ≤9g/dL for males and ≤8.5 g/dL for females

** Missing data were present in 5 persons.
